# Insights into the biochemical and biophysical mechanisms mediating the longevity of the transparent optics of the eye lens

**DOI:** 10.1016/j.jbc.2022.102537

**Published:** 2022-09-27

**Authors:** Roy A. Quinlan, John I. Clark

**Affiliations:** 1 Department of Biosciences, Durham University, South Road Science Site, Durham, United Kingdom; 2 Department of Biological Structure, University of Washington, Seattle, Washington, USA

**Keywords:** eye, cornea, lens, transparency, short-range order, crystallins, aquaporins, intermediate filaments, microcirulation, post-translational modification, deamidation, isomerization, protein condensation, aging, age-related cataract, ARC, age-related cataract, AQP0, aquaporin 0, BFSP1, beaded filament structural protein 1, BFSP2, beaded filament structural protein 2, CL, cataractogenic load, FGF, fibroblast growth factor, GAG, glycosaminoglycan, GRN, gene regulatory network, HMW, high molecular weight, PDGF, platelet-derived growth factor, PG, proteoglycan, PTM, post-translational modification, SRO, short-range order, WIF, water-insoluble fraction

## Abstract

In the human eye, a transparent cornea and lens combine to form the “refracton” to focus images on the retina. This requires the refracton to have a high refractive index “n,” mediated largely by extracellular collagen fibrils in the corneal stroma and the highly concentrated crystallin proteins in the cytoplasm of the lens fiber cells. Transparency is a result of short-range order in the spatial arrangement of corneal collagen fibrils and lens crystallins, generated in part by post-translational modifications (PTMs). However, while corneal collagen is remodeled continuously and replaced, lens crystallins are very long-lived and are not replaced and so accumulate PTMs over a lifetime. Eventually, a tipping point is reached when protein aggregation results in increased light scatter, inevitably leading to the iconic protein condensation–based disease, age-related cataract (ARC). Cataracts account for 50% of vision impairment worldwide, affecting far more people than other well-known protein aggregation–based diseases. However, because accumulation of crystallin PTMs begins before birth and long before ARC presents, we postulate that the lens protein PTMs contribute to a “cataractogenic load” that not only increases with age but also has protective effects on optical function by stabilizing lens crystallins until a tipping point is reached. In this review, we highlight decades of experimental findings that support the potential for PTMs to be protective during normal development. We hypothesize that ARC is preventable by protecting the biochemical and biophysical properties of lens proteins needed to maintain transparency, refraction, and optical function.

Image formation on the retina in the eye requires extraordinary biological specializations. Two tissues in particular are responsible for the optical refraction required to focus images onto the retina. These are the cornea and lens, and both are located in the front part (anterior segment) of the eye (Fig. 1*A*). The cornea (∼550 microns thick) and the lens (4.7–5.0 mm thick) are the two refractive transparent tissues (Fig. 1*A*), which project images onto the retina and its photoreceptors (1, 2, 3). This requires the extracellular corneal stroma and the cytoplasm of lens fiber cells to conform to the same physical laws of transparency and light refraction (4, 5, 6, 7). The importance of their combined optical contribution led to the proposal that the lens (Fig. 1*B*) and the cornea (Fig. 1*A*) should be considered as a single unit in the “refracton hypothesis” (8). With respect to the eye lens, the basis for the refracton hypothesis is the accumulation of diverse, water-soluble, and multifunctional proteins collectively called crystallins. The evolutionary selection of lens crystallins involves gene sharing, their dual function property (9, 10), and their sequence and conformational adaptions to deliver the required refractive index (11, 12). A logical extension of the refracton hypothesis is that the lens and cornea share common biochemical and biophysical properties required for the development and maintenance of a high index of refraction, “n,” *via* protein short-range order (SRO; Fig. 2) and water regulation necessary for transparency (8).

**Figure 1:**
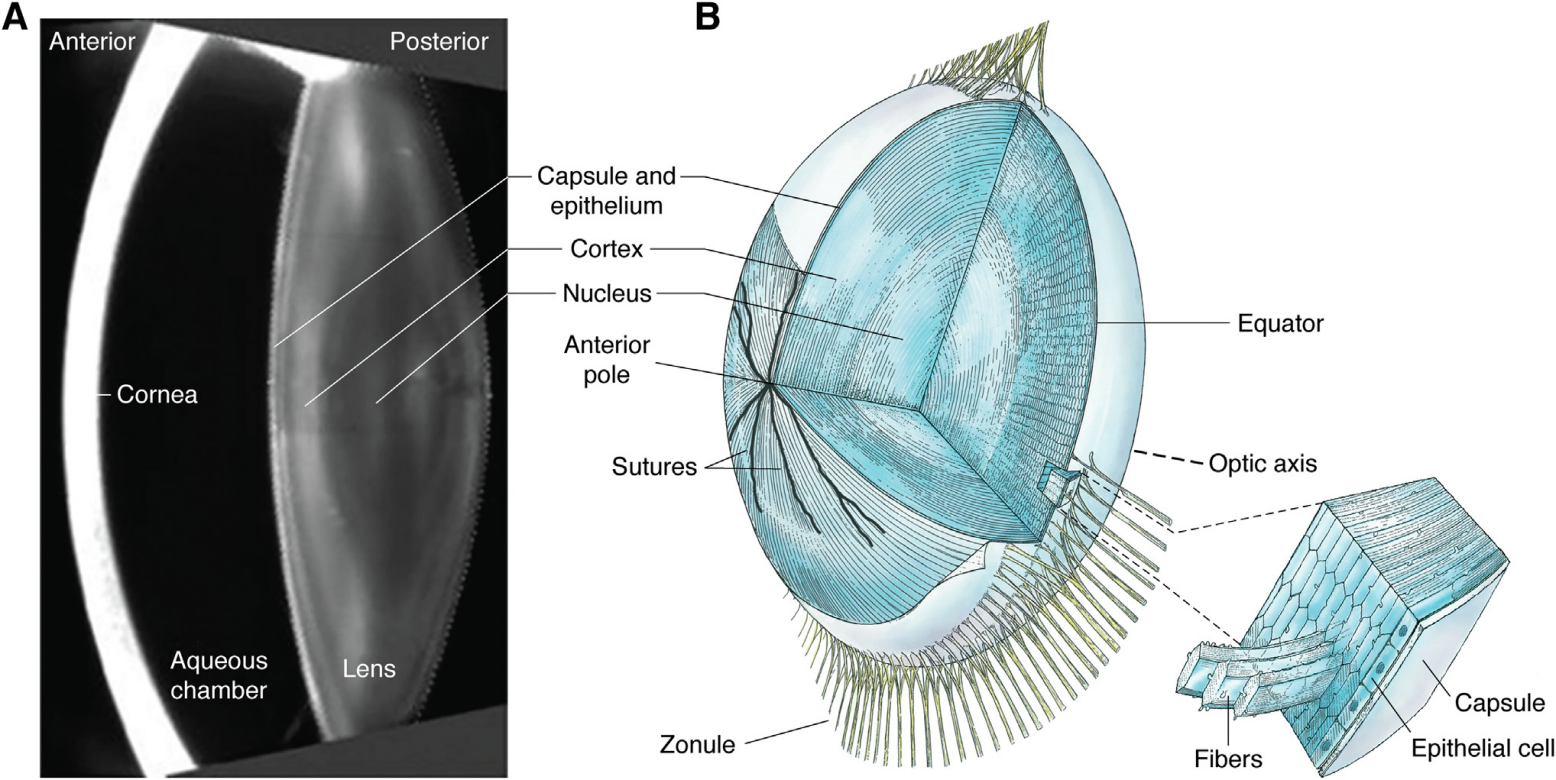
**Vertical section of the anterior chamber of a transparent adult eye and lens anatomy.***A,* the slit lamp image (419) provides an optical section through the anterior segment of the eye. The anterior cornea (*bright curved band*) is separated from the lens by a fluid-filled space (*dark zone between the lens and cornea*), known as the aqueous chamber. The different layers of the lens are visible. A single-layered lens epithelium sits on the inside of the lens capsule, the thickest basement membrane in the human body. The lens capsule and epithelium are seen as a curved bright line on the anterior surface of the lens. Immediately apposed to this surface layer is the cellular mass of the lens, comprising the outer cortical cell layers surrounding the central cell layers of the lens nucleus. The symmetry of the layers results from the coordinated differentiation of cells in the epithelium at the lens equator into lens fiber cells (described in *B*). The variations in the light scattered from the cortical layers are likely because of the different stages of differentiation of the lens fiber cells. Differentiation proceeds from the epithelial cells in the cortical periphery to the central (nuclear) core, so the oldest cells in the human body are in the center of the lens. These cells were produced before birth in the first trimester, so that the cells and proteins of the embryonic lens nucleus are older than the numerical age of the individual. Stability is key to the exceptional longevity of the proteins and cells of the deepest, and oldest, lens layers, where optical function must be maintained for the lifetime of an individual. Cytoplasmic protein concentrations are not only very high to provide help to the required refractive index, n, but transmittance is optimized by short-range order (SRO) and glass-like properties of the lens crystallins. In humans and nonhuman primates, the elasticity of the lens is important for accommodation. The complex gene regulatory networks (GRNs) that are responsible for symmetry, transparency, and longevity remain to be fully identified. *B,* the lens comprises concentric shells of fiber cells surrounding the embryonic nucleus. The oldest cells in the mammal are the lens fiber cells in the central core or embryonic lens nucleus. Lens fiber cells are the progeny of the epithelial monolayer, which generates all new cells in the developing and aging lens. The cortex comprises shells of differentiating lens fiber cells that connect to the anterior and posterior sutures and in cross-section have an iconic hexagonal profile. Differentiation in each growth shell is carefully coordinated, both spatially and temporally, so that the concentric layers are arranged symmetrically around the optical axis. The entire cellular mass is enclosed within the lens capsule, the thickest basement membrane in the human. The resulting optical symmetry is necessary for image formation. Adapted from Ref. (420).

**Figure 2:**
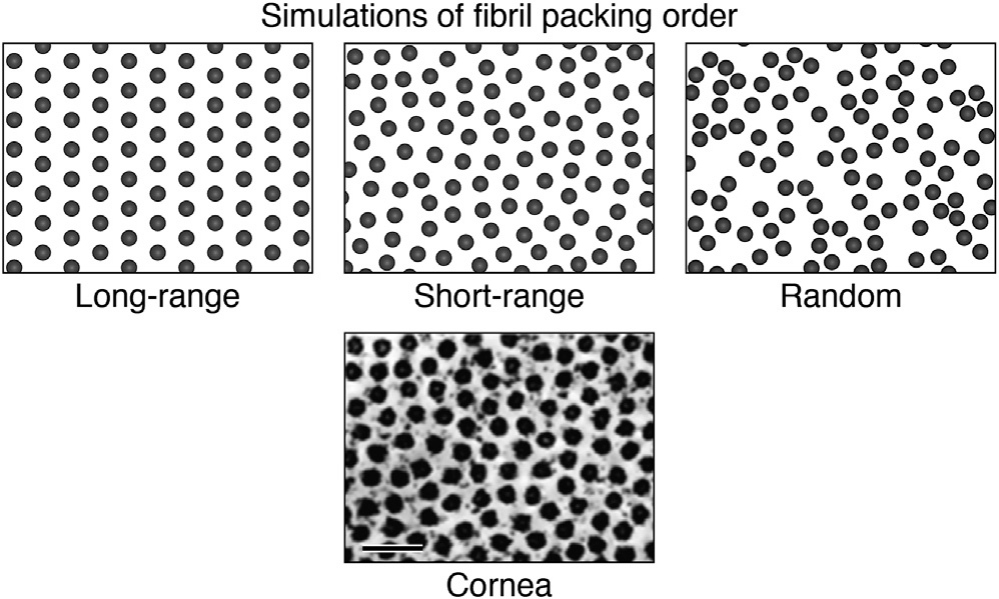
**Short-range order (SRO) and transparency.** An electron micrograph of a cross section of collagen fibrils in the corneal stroma (22) is compared with three simulations of fibril packing order. The concentration of collagen fibrils in the cornea accounts for the high refractive index (n ∼ 1.37), and the fibril packing order accounts for transparency. Random order of fibrils produces opacity. Long-range “crystalline” order results in transparency, but the arrangement of the fibrils has a periodic repeat. In the cornea, fibrils are arranged in SRO, where their spatial positions are variable but nonrandom. Proteoglycans (PGs) maintain the spatial organization of the collagen fibrils that allows transparency (see the text). Hydration of the cornea is carefully controlled to maintain SRO and corneal transmittance. Similarly, lens cell transparency results from SRO in the packing arrangement of the crystallins (bar represents 0.1 micron).

The corneal stroma is approximately 90% of the thickness of the cornea and consists of 200 to 250 extracellular lamellae (sheets) of collagen fibrils embedded in a gel-like matrix of glycosaminoglycan (GAGs) and proteoglycans (PGs) with a small number of keratocytes dispersed throughout (13). The stroma is ∼80% water and ∼15% densely packed collagen fibrils, which increase the refractive index to “n” ∼1.37 (2, 14, 15). In addition to high refractive index, collagen fibrils provide stability to the cornea, preserving its smooth curvature needed for optical quality (16, 17, 18, 19). Theoretically, the concentrated collagen fibrils are consistent with an opaque cornea (Fig. 1*A*; (3, 7)). In the cornea, the spatial arrangement of the fibrils is coordinated by the PGs to limit light scatter. The research on the cornea is extensive, and its transparency is explained by the SRO of the collagen fibrils that optimizes transmittance (Fig. 2; (15, 20, 21)). Similarly, SRO in the lens is thought to account for lens cell transparency (see later). An endothelial cell layer on the posterior surface of the cornea maintains the osmotic balance and hydration necessary for maintenance of uniform optical refraction and image formation required for corneal visual function (Fig. 1*A*; (16, 17, 18, 19)).

In the cornea, PGs and GAGs coordinate the spatial position of the collagen fibrils, largely through hydrogen bonds. The spacing can be altered by increased intraocular pressure or when hydration is not controlled (14, 15, 21). Disruption of the noncovalent hydrogen bonds results in voids or “lakes” that produce inhomogeneities large enough to scatter light ((22); discussed later). Under normal homeostasis, the GAGs and PGs form a resilient scaffold to order the collagen fibril spacing that together resembles a gel-like matrix (15, 20, 21). Just as in the lens, hydration is vital to the development and maintenance of the optical function of the cornea.

## Embryology and SRO in the eye lens

Very early in human embryonic development, a few ectodermal cells, adjacent to the neural plate, swell, thicken, and compress the intercellular space to form a small placode. The placode invaginates and forms a fluid-filled lens vesicle that separates from the surface epithelium that will help form the cornea (1, 23, 24, 25, 26, 27, 28). Migration of neural crest–derived mesodermal fibroblasts behind the remaining ectoderm generates the matrix of the corneal stroma (with its corneal epithelium) directly anterior to the aqueous chamber (Fig. 1*A*).

Adjacent to the aqueous chamber is the lens vesicle, where the posterior epithelial cells exit the cell cycle and elongate to fill the vesicle and form the primary embryonic nucleus of the lens. Figure 1*A* shows the location of the aqueous chamber in the adult eye, and this is the same as in the embryo. A monolayer of epithelial cells remains beneath the thickening anterior capsule ((23, 29); Fig. 1*B*). These anterior epithelial cells are the source of all secondary fiber cells forming symmetric layers that surround the primary lens nucleus (30).

Beginning at the lens equator, the extensive elongation and migration of the secondary fiber cells give rise to mature, millimeter-long, and transparent lens fibers that are never replaced (Fig. 1*B*). The proliferation, migration, and elongation of lens cells occur in the absence of direct contact with surrounding eye tissues, vasculature, innervation, and the immune system. This is due to a thick lens capsule, which defines the physical boundary of the lens. While the differentiation process of the epithelial cells proceeds, water-soluble crystallin proteins are expressed at very high levels, plasma membrane is added as lens fiber cells elongate, all major cell organelles are removed, and an extensive cytoskeletal network forms. The cytoskeleton becomes a scaffold for the condensed crystallins (31, 32) as SRO of the lens cytoplasmic proteins is established in lens fiber cells to optimize lifelong transparency. The massive accumulation of crystallins increases the refractive index, “n,” inside each fiber cell. The ordered close-packed membranes form the iconic hexagonal profile typical of these newly differentiated lens fiber cells (Fig. 1*B*). The embryology of the lens is coordinated with the differentiation of the transparent cornea, as both become integrated into the refracton. These optics adapt continuously to the growing eye (8) over dimensions from 1.0 to 100 mm and over ages that can exceed 300 years without loss of function or cell replacement. Understanding the early embryological basis for the continuous development of the growth shells in the transparent lens is important for two reasons. It shows how the preplacodal ectoderm associated with sensory placodes is modified to generate the refractive properties needed to transform the primordial photoreceptors into a fully functional visual system. It also explains the processes that continue to assemble transparent refractile layers as the lens grows and ages. There is much to learn about the genetics and the gene regulatory networks (GRNs; (33)) of refracton embryology, one of the most remarkable processes in evolution (26, 34).

To summarize, among the important factors needed for the molecular and cellular embryology of the lens are:

(1) Embryologically, the lens begins as a placode of a few elongated and swollen cells at the periphery of the neural ectoderm in the trilaminar embryo. It is unsurprising that many important molecular components are common to both neurons and elongated lens fiber cells (35, 36, 37).
(2) Lens development begins at approximately 50 days embryonic age, and all cells are retained for its lifetime. The cytoskeleton is intimately involved in every developmental stage; in most individuals, this is many decades.
(3) Longevity of optical function is a unique characteristic of the transparent aging lens.
(4) Loss of transparency as manifested by the appearance of cataracts is the leading protein condensation disease associated with aging, far more prevalent than either Alzheimer’s or Lewy body or other aging-associated condensation diseases of the nervous system.

Detailed analyses of the lens epithelium and differentiating fiber cells reveal a more complex cellular organization particularly for the fiber cells ((38, 39, 40); Fig. 1*B*). There are two types of elongating lens fiber cells. A few “straight” fiber cells originate at one pole of the lens but stop short of the opposite pole. Instead, they establish longitudinal meridians known as the lens sutures. Most fiber cells adjacent and parallel to the meridians must curve slightly, with their posterior and anterior ends facing matching parallel fiber cells from the opposing side of each meridional suture line to fill-in each layer or shell. These are known as “s-shaped” lens fiber cells (41, 42). The result is a slight spiral, yet symmetric, organization of lens fiber cells within each cortical layer that could contribute to its accommodative mechanism (43). Whether there is mechanistic and genetic significance to the synchronous and coordinated organization of straight and “s-shaped” fiber cells remains to be determined. The suture lines, however, resemble seams oriented symmetrically about the optical axis at the anterior and posterior poles but arranged to minimize the impact upon the optical efficiency of primate lenses (44). The most important role for the sutures is to facilitate entry of water, ions, small molecules, and nutrients *via* the two lens poles. This is integral to the coordinated internal circulation system within the lens (see later). In the absence of a tissue vasculature, the lens “microcirculation” proves vital to symmetrical lens development and growth (45, 46, 47, 48, 49).

## Transparency and SRO

**Figure 3:**
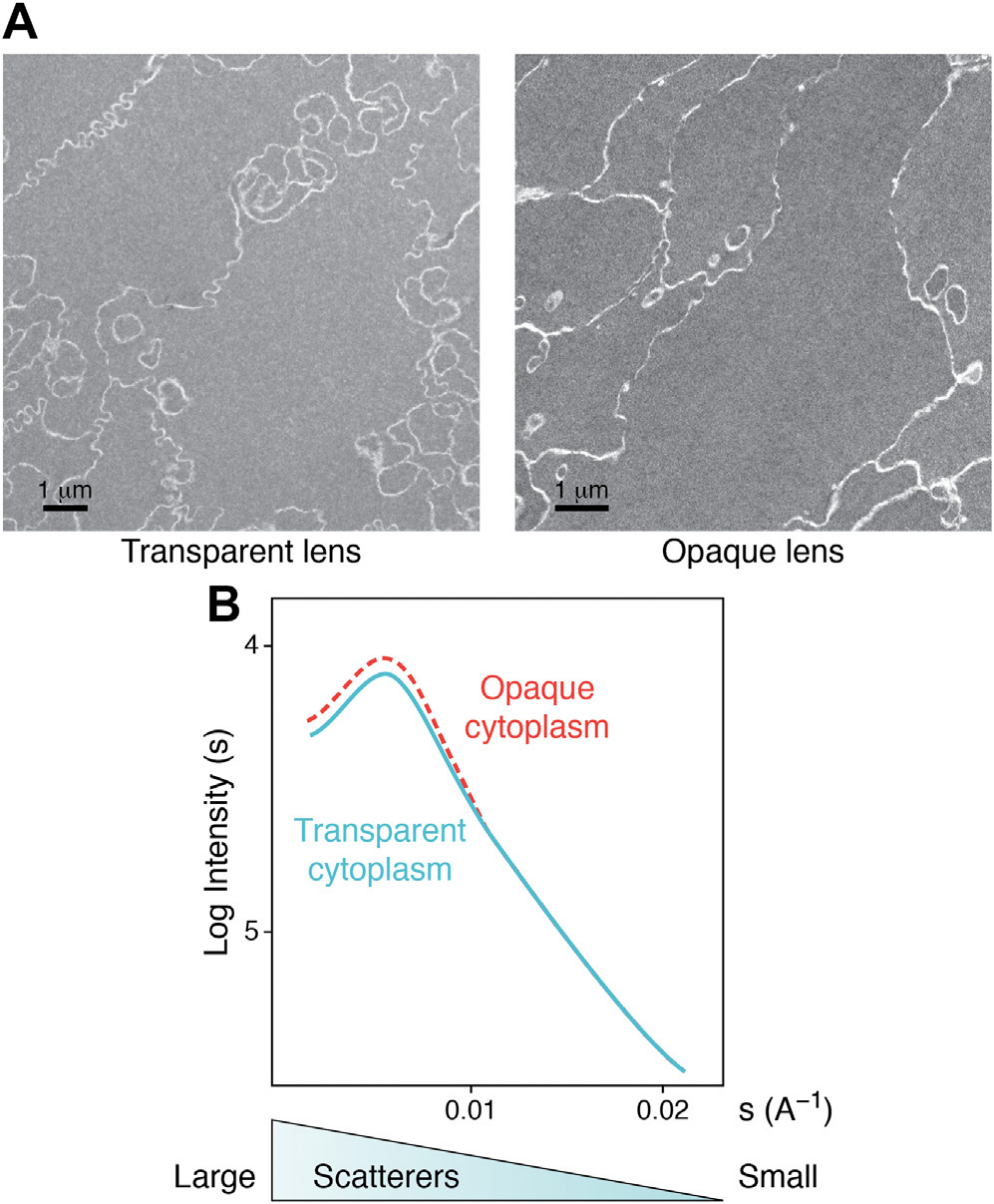
**Comparison of transparent and opaque human lenses by electron microscopy and SAXS.***A,* electron micrographs of cells in a transparent and opaque human lens demonstrate that very small changes in SRO can reduce transparency. Very small differences in SRO between the opaque and transparent lens cell cytoplasm may not be obvious. Quantitative analysis is necessary to determine which cells contain transparent SRO, where destructive interference of scattered light permits transparency, despite the highly concentrated proteins (51). *B,* SAXS from fresh samples of opaque and transparent lens cytoplasm confirms the observations by electron microscopy (*A*). The plots are nearly identical except for a small increase in large scattering components in opaque cytoplasm (*dashed line*). The result demonstrates the impact that very small differences in cytoplasmic SRO can have on lens transparency and can be a model for the earliest reversible stages of formation of pathological cataract. “s” is size; “A” is angstroms; and “I” is intensity (50). SAXS, small-angle X-ray scattering; SRO, short-range order.

Transparency in both the lens and the cornea is facilitated by structural constraints. Instead of ordered collagen fibrils, lens cells contain concentrated solutions of crystallins. In the cornea, weak noncovalent bonds maintain SRO between collagen fibers. In the lens, weak protein–protein interactions between crystallins account for SRO and transparency. In both, destructive interference accounts for light transmission (Fig. 2; (1, 7, 15, 20)). The lens fiber cell cytoplasm is carefully regulated to control the pH, ionic strength, and hydration of the proteins. This happens at the same time as specific post-translational modifications (PTMs) of the surface-exposed side chains of the amino acid residues occur to favor transparency at body temperature. The range of PTMs include racemization, deamidation, oxidation, and phosphorylation. Transparency depends on maintaining the dimensions of protein scatterers below half the wavelength of visible light (400–700 nm). Slight alterations in protein–protein interactions can therefore affect transparency. The difference between SRO in transparent and opaque (cataractous) lenses can be very small as evidenced by data from electron microscopy and small-angle X-ray scattering analyses (Fig. 3, *A* and *B*, respectively; (50, 51, 52)). The data reported in the literature suggest that endogenous mechanisms involving PTMs can help maintain protein solubility to protect and regulate SRO, transparency, and optical function as the lens ages.

## Protein solubility and transparency

Even before the transparent lens was shown to be cellular, early investigations of the lens protein composition recognized the significance of soluble and insoluble fractions in the lens. The insoluble fraction was originally termed albuminoid by Mörner, and the term was adopted by others (53, 54, 55, 56). By the 1970s, opaque water-insoluble fractions (WIFs), often known as the high molecular weight (HMW) fraction (57), were predicted to account for the opacity observed in cataracts (7). These studies determined that the WIF is enriched in cross-linked crystallins (56, 58, 59). The relative proportions of these different fractions were found to be age dependent, and this led to the tacit assumption that cross-linked products in these fractions were the cause of aging cataracts (reviewed in Ref. (60)). The analysis of light scattered directly from intact human lenses made it possible to measure diffusivity of cytoplasmic proteins and calculate the approximate dimensions of scatterers. The calculated dimension of HMW protein scatterers in intact lenses was roughly the same as the dimension of HMW proteins isolated from lens extracts (57, 61).

Historically, it was incorrectly thought that lens transparency results from the precise spacing of cytoplasmic crystallins arranged in long-range crystalline order. SRO is accounted for by crystallin solubility in the absence of protein crystallization or aggregation at very high (up to 600 mg/ml) protein concentrations that are very rarely found in nonlens cells (6, 7, 62, 63, 64, 65). Interestingly though, some lens crystallins retain the enzymatic activities (*e.g.*, lactate dehydrogenase) of the cytoplasmic proteins from which they were derived as a result of “gene sharing” mechanisms (66, 67). Even though the protein concentrations in lens fiber cells can exceed those found in transparent protein crystals used for X-ray crystallography, these multifunctional crystallins remain uncrystallized and in solution inside lens cells.

**Figure 4:**
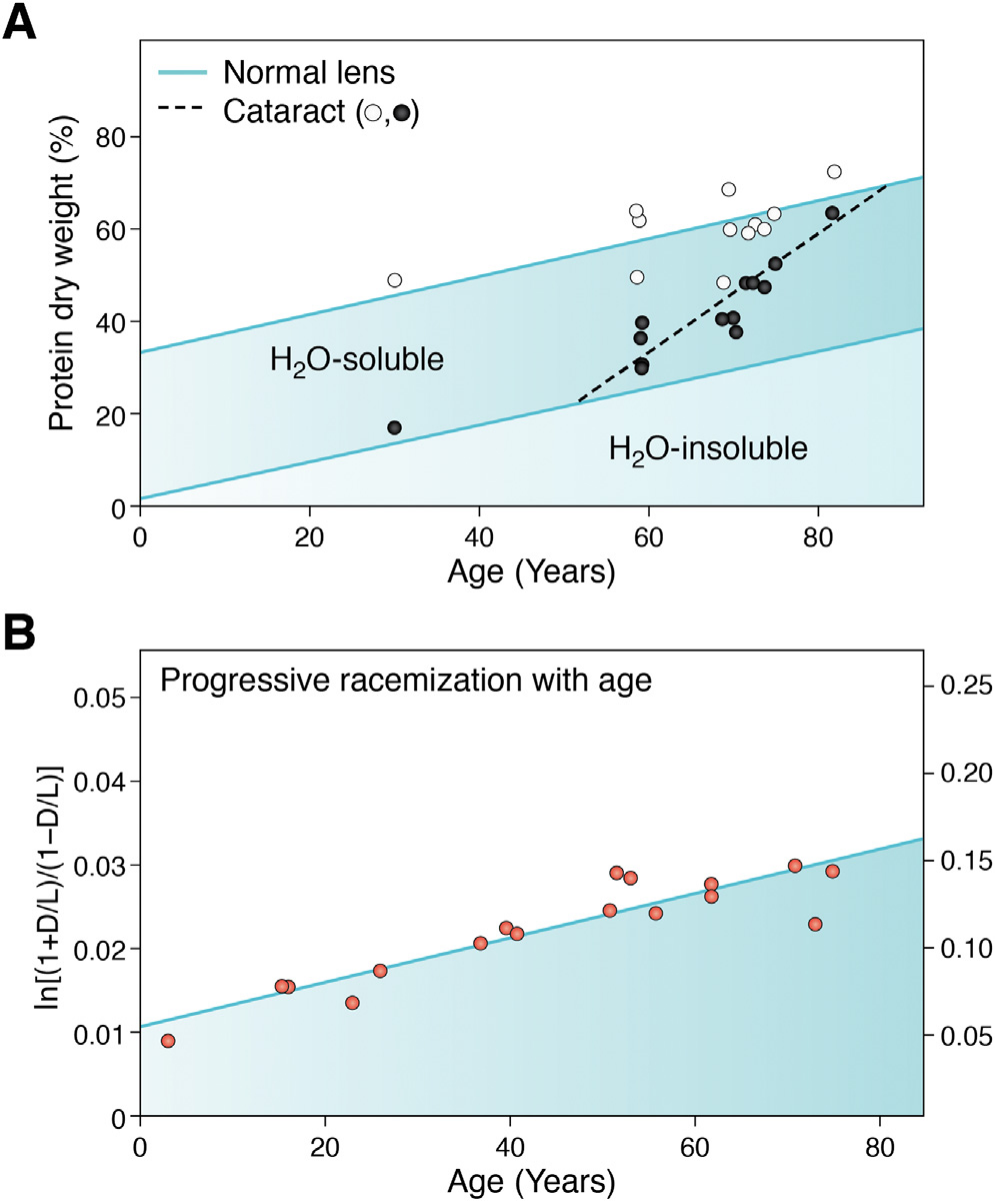
**Progressive insolubility and accumulation of post-translational modifications (PTMs) in cytoplasmic protein with lens age.***A,* total dry weight (%) and water soluble and water insoluble (water insoluble fraction [WIF] = H_2_O insoluble) protein both increase linearly with age in normal human lenses (*solid lines*). These lenses were without cataracts. The WIF increases from approximately 1 to 2% of the total cytoplasmic protein in very young lenses to nearly 40% in very old lenses. *Circles* plot the dry weights for soluble (*open circles*) and WIF protein (*closed circles*) in extracts from cataractous (*opaque*) lenses. The selected cataract samples had extensive opacity in both the cortical and nuclear lens regions. The total dry weights were comparable for both cataract and age-matched noncataractous human lenses. From ∼55 years of age, the WIF of cataractous lenses start to increase at a faster rate (*dashed line*) than in normal age-matched control lenses (*solid line*). As some older transparent lenses have a greater amount of water insoluble protein than samples with severe cataracts obtained from younger individuals, this suggests that the age-dependent insolubilization of lens proteins may not be a measure of cataractogenesis (68). *B,* progressive accumulation of aspartic acid racemization with age (131). Aspartic acid racemization in the central nuclei of 13 group I and II cataracts plotted *versus* age. Fitting the data to a first-order rate equation using least squares (*solid line*) gives *k*_asp_ = 1.29 × 10^−3^/year (*r* = 0.875) or about 0.14%/year. This is identical to the rate observed in the lens during normal aging, although D/L ratios in Asp are observed to be higher in the WIF than the water-soluble fraction (131, 209). This result, among many others, raises the possibility that racemization may be important for the normal development of lens transparency.

Numerous publications allude to a cytoplasmic protein network or gel consistent with the original description of a water insoluble albuminoid fraction in the lens (55, 59, 68, 69, 70, 71, 72, 73, 74, 75, 76, 77). Biochemical and biophysical analyses indicate that the lens cytoplasm resembles a gel, *in vitro* and *in vivo*, and this can account for its glass-like transparency (59, 64, 70, 77, 78, 79). A gel has properties of both a liquid and a solid (80, 81), can stabilize a transparent protein matrix, and is isolated as the WIF. At high protein concentrations, the SRO of the lens crystallin stabilized in a gel can decrease light scattering in comparison to that predicted for proteins acting as independent scatterers (5, 7, 69, 75). Whilst close packing of lens cytoplasmic proteins can decrease light scattering, protein crowding is often associated with the formation of unstable oligomers, fibrils, aggregates, or gels (82, 83, 84, 85, 86). Lens fiber cells are exposed to a variety of stresses, including hypoxia, high ionic strength, changes in osmotic pressure, decreasing pH, and high protein concentration (87, 88, 89), as the HMW and WIF (Fig. 4*A*), and PTMs (Fig. 4*B*) increase progressively with age. Under these crowded conditions, SRO and transparency are retained even as proteins slowly become insoluble (64, 90, 91).

## Cataractogenic load hypothesis and its implication for lens transparency

**Figure 5:**
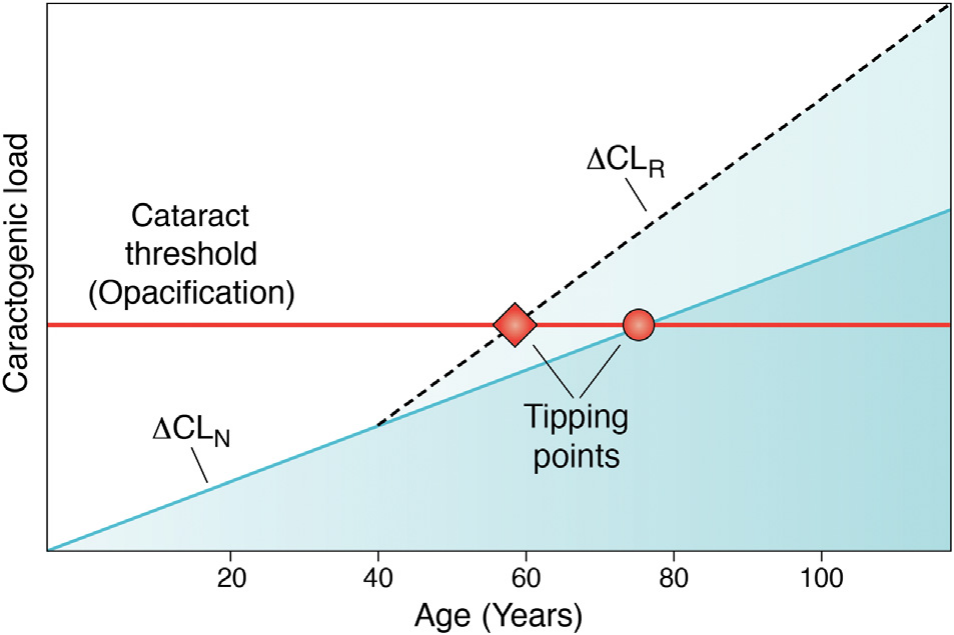
**Cataractogenic load (CL) hypothesis of lens aging.** During the development of transparency, the post-translational modification (PTM) rate (ΔCL_N_) is slow and progressive. Late in life, at the intersection of CL and the initial stage of opacification (*horizontal red line*), a tipping point (*red dot*) occurs. This is often observed in a slit lamp examination of the eye lens in an individual. When cataractogenic conditions develop, the ΔCL_R_ (*dashed line*) increases and the tipping point (*red diamond*) occurs at an early age for any single eye. A corollary to the CL hypothesis states that a decrease in ΔCL can protect transparency and extend the longevity of the lens function. The CL hypothesis (104) is based on concepts discussed by C. Kupfer, the first director of the National Eye Institute (142) and by G.R. Merriam, A. Szechter, and B.V. Worgul (92, 109, 421, 422).

The cataractogenic load (CL) hypothesis acknowledges the collective effect of the progressive modification of lens cell constituents (proteins, DNA, and lipid membranes) that occur with age and with environmental, nutritional, metabolic, and genetic stresses upon the lens. The primary risk factor for reduced optical function and loss in lens transparency is age (1, 92, 93, 94, 95, 96, 97, 98, 99, 100). Some of the oldest cells in the body, and therefore the oldest proteins (101, 102, 103), are in the eye lens, making the lens an excellent model for research on molecular and cellular aging. In systematic studies of aging lenses, proteins, DNA, and lipid membranes are modified with age (104, 105, 106, 107). The progressive effect on transparency can be characterized as the CL, eventually leading to aging cataracts (Fig. 5; (104)). The CL hypothesis implies that the progressive modification of lens constituents may be protective during development and lens aging, prior to any appearance of insult-induced or aging cataracts.

Based on the multiple effects of ionizing radiation on lens cells and their constituents, a model is proposed to recognize the potential protective impact of PTMs accumulated with development and aging (104). Ionizing radiation is considered one of the most effective ways to accelerate lens aging *in vivo* and *in vitro* (104, 108, 109, 110, 111). Few cataract models have been studied more than the lens opacities resulting from ionizing radiation exposure, in part not only because of their similarities to opacification with age but also because of the exposure of individuals involved in air travel, space exploration, medical imaging, and intervention therapies (104, 112, 113, 114, 115). Irradiated lenses show altered metabolism, PTMs, oxidative and osmotic stress, increased insoluble protein and protein aggregation, decreased pH and ion imbalance, and light scattering (116, 117, 118, 119). These published data provide insight into the basis for molecular and cellular longevity in the aging lens (112) and the mechanisms of aging on the sequence of molecular changes that occur in aging cells and tissues (93, 95, 96, 99). Extensive research on radiation-induced cataracts led to a novel interpretation (the CL hypothesis) for molecular and cellular longevity in the lens (Fig. 5) and identifies the potential role of PTMs in protecting lens transparency and optical function (104, 120).

## PTMs in lens proteins and transparency

Even before birth (121, 122, 123), lens proteins start to accumulate PTMs (124, 125, 126). In 1942, G.L. Walls observed that “The lens is unique among the organs of the body in that its development never ceases, while senescence commences even before birth” (127, 128). The tacit assumption has been that the PTMs associated with lens development are eventually responsible for cataracts (123, 126, 129, 130). A different interpretation is that these PTMs may protect and maintain SRO, transparency, and symmetry as the lens ages (see later). The most abundant PTM by an order of magnitude in aged transparent human lenses is racemization, particularly of Asp/Asn and Ser (l- to d-isomer; (131)) followed then by deamidation (132, 133, 134, 135, 136). These are common modifications known to proceed through a succinimide intermediate with known kinetics and reaction products that are often resistant to spontaneous biochemical degradation or proteolytic cleavage in living cells (137, 138, 139). To categorize PTMs with protective or damaging effects on lens function, datasets that quantify differences in deamidation rates within crystallins need to be characterized by age and transparency. This also applies to levels of racemization and isomerization, which potentially perturb protein structure greater than deamidation alone (131, 132, 133, 134, 135, 136, 137, 138, 139). In long-lived biological cells and tissues, racemization and deamidation can be an accurate measure of age, comparable to radiocarbon dating (140). While their resistance to degradation or proteolysis can account for their accumulation with age, there may be a threshold for their protective effect *in vivo*. Perhaps, such a threshold could equate to a tipping point, which could occur when the balance between favorable and unfavorable interactions is disrupted, leading to an increase in the rate of change in CL (Fig. 5; (104)). This threshold could occur prior to changes in the PTM landscape of lens proteins for an individual lens (141). It should be noted that the PTM profile of aged transparent lenses is reported to show little variation between different people (141), suggesting that a PTM signature for aging transparent lenses could be produced. This will help identify, for example, which succinimide intermediates are protective against loss of function in cell and molecular aging. The predicted tipping point may coincide with a collective change in CL (141) when the predominant size of scatterers approaches or exceeds λ/2, where λ is the wavelength of visible light (380–740 nm; (1)) and initiate loss of transparency. It could be challenging to generate a PTM signature for age-related cataract (ARC) given the variation between different individuals. Quantitative data are needed to determine the importance of the major age-related PTMs in maintenance of lens transparency and optical function. It should be noted that the CL hypothesis is consistent with conceptual approaches for effective therapeutic protection against clinical cataract formation (69, 92, 142, 143).

Considerable effort has gone into defining PTMs involved with transparency and refraction for eye tissues. In the study of the cornea, there is agreement on the importance of PGs and GAGs in the control of the spatial order of the collagen fibril matrix that is necessary for SRO in the stroma (14, 19, 21, 144). SRO and transparency in the cornea are better understood, and similar biochemical interactions may also control SRO and transparency in the lens (1, 8). For both systems though, the details of the changes in protein–protein interactions resulting from PTMs during aging in the establishment of SRO in differentiation of transparent lens fiber cells, remain to be characterized fully (6, 70, 90, 114, 145, 146, 147).

If a single PTM were responsible for SRO and transparency in the lens, then its characterization and spatial distribution would be a relatively simple task (*e.g.*, Fig. 4*B*). Thus far, no direct link between a single PTM in an individual lens protein and the onset of cataracts has been found, but some potential associations have been identified (135, 139, 148, 149, 150, 151). PTMs are accumulated in many lens proteins with age (126, 132, 135, 139, 152, 153, 154, 155, 156). These previous studies were careful to identify the aging-associated PTMs (123, 126, 129, 130), which can then allow the spatial and functional characterization of specific protein–protein interactions that control cytoplasmic SRO and cellular transparency to be identified.

Global approaches have been used to study deamidation as a major PTM of lens proteins (135, 157). Deamidation of Asn and Gln residues is identified in proteins isolated from human cataractous and aged lenses. It is estimated to account for 66% of the total PTMs tabulated when water-soluble fractions and WIF were analyzed (134). Modern technological advances in mass spectrometry will continue to develop our appreciation of PTM abundance and protein profiles in the aging lens (158, 159). To follow dynamic changes in lens transparency, a measure of the collective and diverse protein interactions is needed, matched to the age-related PTMs for lens proteins. Such analyses would support the delivery of an effective and protective therapeutic capable of delaying the (age-related) loss of transparency.

Crystallin PTMs start to accumulate even whilst the fetus is in the womb (121, 122, 123, 136, 156), long before any loss of transparency is observed in an aging lens. At the other end of the age spectrum, centenarians have a lower-than-expected incidence of ARC (160, 161). Although it might be expected that PTM would be progressive and similar in both lenses, ARC is not always bilateral (162, 163, 164, 165), and many factors play a role in the loss of transparency (165, 166). It is important to acknowledge that life span and aging are not necessarily the same even for monozygotic human twins (167, 168, 169), as lifestyle is a potential modifier (170, 171, 172). It is, however, well established that crystallins are extensively modified during normal aging and in cataracts (122, 126, 130, 134, 154, 173, 174, 175, 176, 177, 178). The use of mass spectrometry has unambiguously identified deamidation as the cause for the increase in acidification of the lens crystallins in early studies of the human lens (179, 180, 181, 182, 183, 184). Many of these studies analyzed deamidation in either purified crystallins or for a specific amide for all lens crystallins (129, 130, 175, 176, 185, 186, 187). When an eye lens is homogenized and the proportion of the various protein fractions (*e.g.*, water soluble, HMW, WIF) is analyzed, increasing proportions of the proteins from the innermost and older “nuclear” region are found in the WIF in an age-dependent manner (Fig. 4*A*; (188)). The long-standing and widely held hypothesis is that the accumulation of crystallin PTMs is the cause of this insolubilization and subsequent cataract formation (139, 154, 189, 190, 191, 192). The experimental evidence suggests that we have been too quick to judge the role of PTMs in the loss of transparency and cataract formation (153, 193, 194, 195, 196, 197, 198, 199) rather than being connected to the formation, maintenance, and retention of SRO during lens aging. Lens protein PTMs are kept under constant review (139, 141, 189, 200, 201, 202). Experimental data consistently demonstrate that most lens cytoplasmic protein PTMs identified in embryonic and young lenses can contribute to the development and maintenance of lens transparency (139, 154, 189, 190, 191, 192).

We suggested previously (104, 120, 125), and others (203) have similarly speculated recently, an alternative hypothesis regarding the role of lens protein PTMs in lens transparency and optical function. When crystallin PTMs were first being identified, those associated with normal aging were included with those associated with cataracts (123, 129, 130). In the CL hypothesis, we propose that many lens protein PTMs have an active protective role in preserving transparency and optical function (Fig. 5). PTMs, such as deamidation (123, 129, 136), proteolysis (139), and disulphide bond formation (156), are necessary for the early development, establishment, and maintenance of SRO in the lens. It has been suggested previously that the proteolytic removal of the N-terminal extensions of β-crystallins has a potential stabilization/protection role (139) that facilitates crystallin SRO. The disulphide bonding and exchange that occurs because of the oxidoreductase activities of lens crystallins is also considered to be protective and could be a stabilizing influence in crystallin gels (201, 204). Others have recognized that “crystallin PTMs act in a complementary and synergistic manner, potentially coupled with some redundancy, such that they do not have a prejudicial effect on crystallin solubility and interactions (197).” We suggest that such aging-associated PTMs can be protective rather than passive in this role. Indeed, we note that the PTM profiles are different in the WIF, HMW, and membrane lens fractions (134, 136, 141, 153, 154, 155, 158, 205, 206), so their PTM landscapes need to be characterized and their age-dependent profiles determined. There is evidence for γD-crystallin that deamidation is not a driver for cataract formation (207). To illustrate the extent and significance of the accumulated crystallin PTMs, every polypeptide in the transparent optically functional lens of a 60 year old contains a minimum of several d-amino acids as a result of racemization (132), which is thought to be the most common PTM during aging ((129, 139, 184, 208, 209); Fig. 4*B*). Such data argue for a protective rather than destructive role for some, if not most, of the protein PTMs in the aging lens supported by the fact that the PTM landscape remains surprisingly consistent between different individuals (141). This acknowledges that PTMs present during early development (126) contribute to the stabilization of, rather than the compromization of the SRO as the lens ages *in vivo* (200, 207, 210). This premise makes sense from the perspective of maintaining optical function for decades, sometimes over a century, despite the fact that PTMs are continuously accumulated over this period in the human lens (141). Diverse stabilizing interactions between cytoplasmic proteins in the absence of cellular adaptation and protein replacement, deliver molecular and cellular stability for functional longevity (211, 212, 213). It is worth noting that cataract formation changes the PTM signature of aged transparent lenses (141). For example, oxidation-mediated PTMs are associated with cataracts (129, 193, 195, 196), and the data suggest that these PTMs may result from crossing the CL tipping point once protein aggregation is initiated. Protein aggregation, lens opacification (Fig. 5), and dramatic alterations of the PTM landscape are a consequence (141).

Aspartate racemization of lens proteins increases with age (Fig. 4*B*) and with the accumulation of insoluble proteins (WIF) and HMW aggregates (214, 215, 216) (Fig. 4*A*). Without a quantifiable relationship between PTM and transparency, it is reasonable to consider that insolubility, as represented by the WIF and HMW aggregates, could function in the stabilization of lens proteins. Rather than causing cataracts (121, 192, 197, 198, 217, 218), WIF and HMW may be a measure of the gel state in the cytoplasm that helps maintain SRO as cytoplasmic protein concentrations increase during development of transparency (197). The fact that the reported PTM profiles for the WIF, HMW, and the water-soluble fraction are not the same (134, 136, 141, 154, 155, 158, 205, 206) suggests that PTMs have a function in the separate fractions, rather than a passive role as suggested recently (197).

It is to be expected that some PTMs will be detrimental to protein SRO and lens transparency. In examples of a deamidated asparagine or glutamine residue where a single change in charge is exposed on the protein surface (129, 184, 208), an increase in attractive interactions favors protein aggregation and light scattering, at least *in vitro* (135, 190, 217). When the deamidation is buried within the crystallin tertiary structure, loss of conformation can occur, leading to protein unfolding, aggregation, and light scattering (219). What if the behavior *in vivo* is different? Weak and noncovalent interactions are known to crosslink and stabilize lens crystallins (145, 146, 212, 220, 221) similar to the effect of PGs in the corneal matrix that maintain the SRO of collagen fibrils in corneal stroma (22, 222). Stabilization of SRO permits destructive interference of scattered light, favoring light transmission and image formation in the eye (2, 19). When increased hydration disrupts the SRO in the corneal stromal matrix, light scattering increases resulting in corneal opacification. In the lens, an initial increase in light scattering can be observed *in vivo* using quantitative and dynamic spectroscopic instrumentation as the lens ages (70, 79, 223, 224, 225). This may be a very sensitive measure of the tipping point in the CL hypothesis (Fig. 5).

Protective crystallin PTMs include acetylation, carbamylation, and ascorbylation, and these support the concept that crystallin PTMs can maintain SRO and lens transparency (226, 227, 228). As many as 19 phosphorylation sites are reported on alpha crystallins in an adult human lens (229, 230, 231). While crystallin phosphorylation varies dramatically during development and aging, the change in charge may initially stabilize SRO in the lens cytoplasm. As in the CL hypothesis, when protein conformation and protein–protein interactions are altered, loss of transparency can occur (Fig. 5). Disulfide formation, advanced glycation end products, and PTMs as a result of reactive oxygen species that accumulate with age can then crosslink proteins altering protein–protein interactions, adding to the CL, and hastening the arrival of a tipping point (Fig. 5). Once formed, however, covalent crosslinks are not easily reversed although disulphide exchange *via* the oxidoreductase activities of lens crystallins means this PTM is dynamic within the protein complex (201, 204, 232). Over time, the cumulative effect of covalent crosslinks can disrupt SRO and cause irreversible light scatter. No therapeutic method successfully reverses the impact of covalent interactions between molecular and cellular constituents *in vivo*, making a surgical intervention the only treatment strategy currently available.

It is important to note that weak and noncovalent interactions can be reversed by endogenous protective mechanisms. The role of α-crystallin, as a small heat shock protein and chaperone, is an additional protective mechanism helping to limit aggregation within the crowded protein environment of the lens cytoplasm (91, 233, 234, 235, 236). Aggregation is prevented by α-crystallin, the resident small heat shock protein complex of αA-crystallin and αB-crystallin (237). Aging compromises the chaperone function of α-crystallin (238, 239, 240, 241, 242) and permits light-scattering aggregates to increase with age (243). Systematic studies of crystallin PTMs that accumulate over the lifetime of a lens are needed to determine which PTMs protect transparency and SRO, and which drive protein aggregation in lens fiber cells. Modern quantitative dynamic laser scattering of lenses *in vivo* correlated with mass spectroscopic analyses (both spatial and temporal) at selected time points will clarify the relationship(s) between important optical and biochemical events in development and aging.

## Metabolic adaption and lens transparency

Although the lens and its cells are isolated from vasculature and lymphatics of the eye, it is still capable of tissue repair (244) and inflammatory responses when damaged (245, 246). Unlike other aging epithelia, lens function is protected against programmed cell death, replacement, cellular immunity, and inflammation through mechanisms known as anterior chamber immune-associated deviation (247, 248). The aqueous humor includes immunosuppressive factors. Taken together, these are unique mechanisms that protect lens transparency from being compromised by trauma, inflammation, or infection (249).

During lens differentiation, metabolism in lens fiber cells is limited to pathways promoting and protecting transparency (78). The primary metabolic pathways in mature lens fiber cells are anaerobic glycolysis, hexose monophosphate shunt, pentose phosphate pathway, and the polyol pathway. Enzymes in these primary metabolic pathways are selected to balance ion homeostasis, hydration, maintain protein solubility, and function in the absence of membrane-bound organelles. In mature lens fiber cells, ATP, GSH, myo-inositol, NADPH, and polyols are needed to preserve protein solubility and SRO and to protect against light scattering and opacity. Several publications summarize the importance of lens metabolism to the maintenance of protein solubility, stability, structure, symmetric microcirculation, and resist oxidative and osmotic stress (87, 93, 249, 250, 251, 252, 253, 254, 255, 256, 257).

## Lens microcirculation and the importance of hydration to the refracton

As mentioned previously, hydration is vital to the maintenance of transparency in the refracton, the optical unit of the eye. This is true of the corneal epithelium where there are physical barriers to water and in the corneal endothelium, which is enriched in water pumps (258). Abnormal hydration of the corneal stroma can occur when inhibition of metabolism results in fluid-filled spaces to cause large changes in SRO and light scattering. The opacity is reversible if caught in the early stages (22, 259, 260).

**Figure 6:**
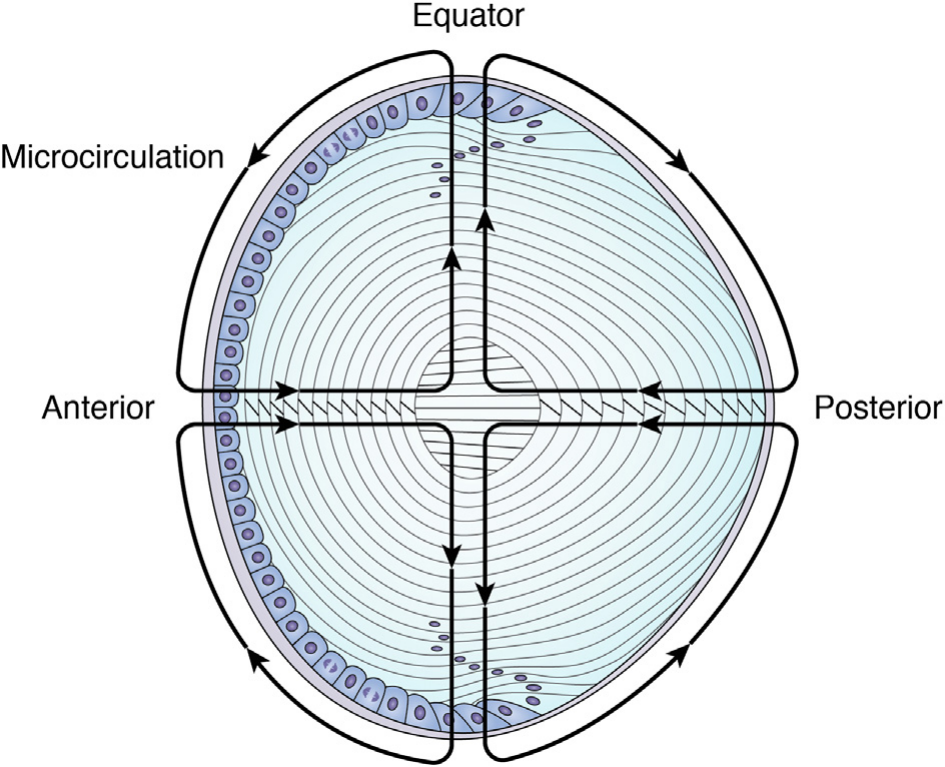
**Microcirculation shown for a lens cross section.** The shells of mature fiber cells are in the optic path of incoming photons. In the absence of vasculature, homeostasis is maintained throughout the lens by the symmetric circulation of ion and fluid fluxes entering the lens at both the anterior and posterior sutures and penetrating to all cells *via* the extracellular space (*inward*→←). Membrane channels and gap junctions transport fluid and ions peripherally to exit at the lens equator (*outward*↑↓). The spatial distribution of channels is controlled to support optical symmetry during the formation of highly refractive and transparent mature fiber cells. Adapted from Ref. (45).

In lens fiber cells, the highly concentrated crystallins require controlled hydration to maintain their transparency ((261); Fig. 6). One of the earliest mechanisms identified as a cause for the loss of lens transparency is osmotic stress resulting from altered metabolism and channel dysfunction. This prevents the unique lens microcirculation system (see later) from balancing the osmotic pressure of highly condensed proteins across fiber cell membranes (2, 262, 263, 264, 265, 266). While such osmotic stresses can be caused by uncontrolled diabetes, they are reversible if treated early (244, 267, 268). More recent studies suggest that syneresis (the separation of water from a protein solution) is a mechanism for an increase in free water when cytoplasmic protein networks collapse and light scatter is increased (75, 261). The theory of syneresis acknowledges the gel-like nature of lens cytoplasm because syneresis is observed in protein gels (74). The loss of transparency resulting from syneresis in lens cytoplasm is analogous to the appearance of fluid-filled spaces in the opaque corneal stroma (see previous one). It must be emphasized that the very high concentrations of proteins in lens fiber cells required for the high index of refraction can generate substantial osmotic pressure. In studies of lens syneresis, osmotic pressure was not determined, and mechanistically, syneresis may correspond to an osmotic stress. A prominent metabolic pathway, the polyol pathway, is thought to respond when osmotic conditions become unbalanced in lens fiber cells. Therapeutics that control the polyol pathway are currently in use in diabetic animals as an effective regulator of osmotic cataracts associated with diabetes (269).

In humans, the successful maintenance of lens hydration depends on a unique and complex microcirculation system of water and ions (Fig. 6). It is expected that the microcirculation of fluid, ions, and nutrients to supply the lens fiber cells is symmetric in order to support optical symmetry (48, 270). In the lens microcirculation model, fluid enters the lens at both the anterior and posterior sutures to penetrate uniformly deep to the nuclear cells *via* the extracellular space (262, 271). Fluid exits at the equator using membrane channels and gap junction (Fig. 6). These extraordinary electrophysiological properties were first observed in the 1970s (272, 273, 274). More recent electrophysiological studies identified small electric fields that can drive bidirectional migration with the function of fibroblast growth factor (FGF) and platelet-derived growth factor (PDGF) in the differentiation of the symmetric shells of lens fiber cells ((275, 276); see later). Sensitive methods of light scattering *in vivo* can be correlated with recent MRI data from the systematic study of lens hydration. This can provide new insights into the functional relationship between transparency and microcirculation during development and aging (270, 277).

## Lens symmetry and transparency

Lens symmetry starts in the epithelium and is reinforced during differentiation of the secondary fiber cells to generate the refractive, transparent, and long-lived lens fibers (Fig. 1*B*). As synchronized cell elongation begins, differentiating lens fiber cells assemble radial growth shells (40, 41, 42). The new growth shells surround previous layers of differentiating fiber cells, increasing the anterior–posterior thickness and lateral diameter of the lens (267, 278). The differentiating fiber cells are extensively connected through a network of organized cell membranes containing channel proteins, and the expansion of their surface area featuring membrane projections, microridges, and modified microvilli (78, 279, 280, 281, 282). Electrophysiological and dye diffusion studies demonstrate that the cells in each layer of differentiating fiber cells are closely coupled (Fig. 1*B*; (36, 79, 265, 283, 284, 285, 286, 287)). The high degree of intercellular connectivity within the growth shells (36, 288) is necessary for the spatial continuity of transparency, and the symmetric gradient of refractive index across the diameter of the lens (213, 289, 290, 291, 292). The membranes are enriched in membrane proteins and sensors, including connexins, aquaporins, the transient receptor potential cation channels, TRPV1 and TRPV4, as well as cell adhesion molecules (282, 289). In the absence of vasculature, the expanded surface area of the plasma membranes is crucial to the lens microcirculation (46, 260, 293, 294, 295, 296). Electrophysiologists often refer to the lens as a “syncytium” because of its very high electrical coupling (297), but this is a misnomer because the lens consists of distinct cells. While it is correct that the lens membranes form a highly symmetric network of interconnected phospholipid bilayers, similar to cardiac muscle and other excitable smooth muscle (298), they are not multinucleated, a characteristic of a biological syncytium. In fact, nuclei and all membranous organelles, including the endoplasmic reticulum, mitochondria, and Golgi apparatus, are removed during fiber cell differentiation, an advantage for transparency, because whole and fragmented organelles are large enough to scatter light and interfere with image formation (79). The coupled symmetric shells of lens fiber cells and their constituent proteins appear to be stabilized to protect optical function for a lifetime (40, 41, 42). While the significance of symmetry for optical function is obvious (6, 40, 278, 288, 299, 300, 301, 302), continued research into the genetic pathways for coordinated differentiation and establishment of symmetry as well as SRO *in vivo* is under-researched as an emergent property of lens fiber cells and their tissue-level order. The intersecting developmental, genetic, and molecular pathways for synchronization, longevity, and symmetry in optical function need to be better understood (25, 29, 303, 304). Without symmetry, a ubiquitous concept in science (305, 306, 307), image formation on the retina in the eye would not be achieved (40).

Biochemically, the establishment and maintenance of symmetry to lens function is linked to the differential effects of growth factors and channel protein expression during development of the microcirculation system in the lens (29, 45, 262, 270, 277, 299, 308, 309, 310, 311). While the importance of nutrition in lens transparency is also well known, the uniform spatial organization of fluid flow and its role in lens symmetry receives little attention (166, 300, 312, 313, 314). To establish and maintain the optical symmetry necessary to project images onto the retina, the differentiation of lens cells in their radial layers to form symmetric shells of interconnected fiber cells (314, 315) needs to be carefully coordinated both spatially and temporally. The formation of symmetric and concentric spherical layers of elongated denucleated fiber cells in the lens is complex (40), starting with the synchronous proliferation and migration of epithelial cells located in the most distal region of the lens epithelium (118, 300, 316, 317). These are found in the germinative and transitional zones of the epithelium, and their synchronized differentiation is initiated in response to proliferative factors including FGF, insulin-like growth factor, bone morphogenetic protein, and PDGF (283, 284, 285, 286, 293, 300, 311, 313, 316). It appears that the remarkable symmetry, transparency, elevated “n,” and dry weight may depend on pulses of PDGF (283, 287, 294, 295, 296, 297, 301, 302) that can synchronize DNA synthesis and mRNA production with the cell cycle (294). It would be unsurprising if the transcriptional bursts reported recently in lens fiber cells correspond with the cell cycle and pulsatile growth factor stimulation as lens symmetry and optical function develops (298). It could be expected that the transcriptional bursts are synchronized and regulated by growth factors in the coordinated development of symmetry, transparency, and optical function and correspond spatially with the proliferating bands of cells in the epithelium (288, 294, 300, 301, 318, 319). Evidence of connexin permeability to important signaling molecules is consistent with a role for microcirculation in a mechanism for establishing and maintaining optical symmetry (296) and for the coordinated response of the lens to spatially restricted stresses (320, 321). Recent research on lens water and ion channels leaves little doubt regarding the importance of the microcirculation to the optical function of the lens (270, 277).

## The importance of lens fiber cell membranes and their associated cytoskeleton to lens transparency and optical function

**Figure 7:**
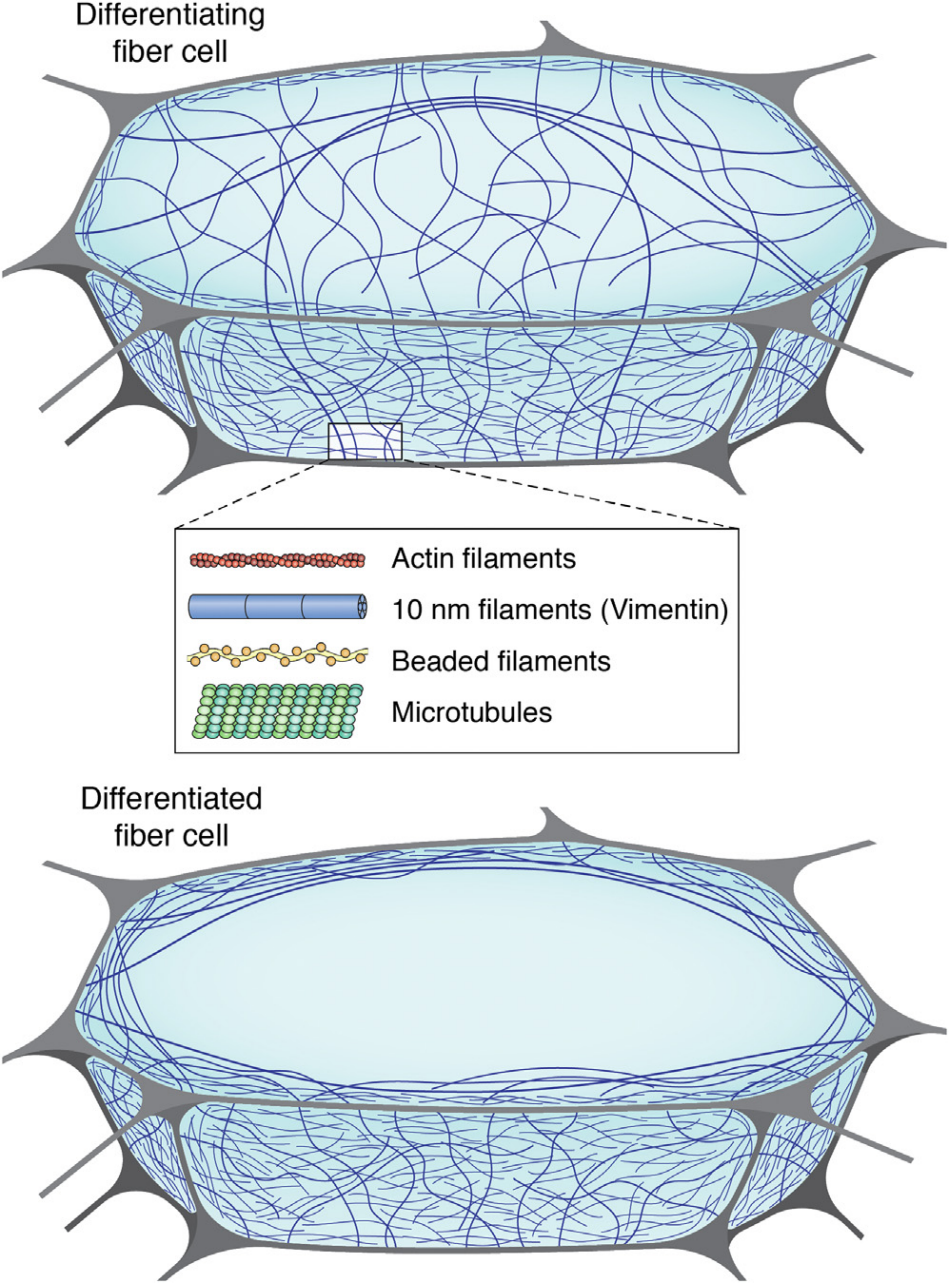
**The cytoskeleton in the lens fiber cells of the outer differentiating compartment (*top*) and the differentiated cortical fiber cell (*bottom*).** The lens cytoskeleton has multiple filamentous components as shown in the schematic of a cross-section of individual fiber cells: beaded filaments, intermediate filaments, actin, both stable and dynamic microtubules, and their associated proteins. During the differentiation from epithelium to mature lens fibers, cells migrate, elongate, and increase the expression of water-soluble crystallins. As the “n” increases dramatically, the cytoskeleton stabilizes the elongated shape of the membranes and serves as a scaffold to organize crystallin short-range order (SRO). Once SRO is established in cortical lens fiber cells, the cytoskeleton redistributes from the cytoplasm to the plasma membranes at the cell periphery and thereby helps to stabilize the expanding phospholipid bilayer, into surface projections, and microridges.

The lens cytoplasm abuts the fiber cell membranes that are lined with a dense cytoskeletal matrix driven in part by the condensation of the cytoskeleton onto the membrane. The membranes also help maintain the very low oxygen tension and the anaerobic environment in the center of the lens because of their high cholesterol content (322, 323, 324). While there are differences in membranes during development and aging, all observations are consistent, and differences in “n” between the cytoplasm and the membranes cancel out to maintain transparency (72, 77, 78, 325, 326). Small changes in refractive index are observed as lens birefringence and are due to both the cytoskeleton and the age-related changes in the cytoplasm (Fig. 7). The lipid bilayer is compressed, reducing plasma membrane thickness to cancel out differences in “n” between the cytoplasm and the membranes, and optimize transparency (72).

Membranes and the associated cytoskeleton are fundamental to the lifelong function of the lens, as the membranes control water, ion fluxes, and transport of antioxidants and metabolites into and out of the lens fiber cells (48, 277, 296). The cytoskeleton is anchored to and stabilizes the cell membranes (124, 327, 328). Cytoplasmic proteins partition along the lens membranes as the fiber cells mature (68, 214, 216, 230, 329, 330, 331), and protein PTMs accumulate (155, 159, 230, 332), affecting lipid organization (333, 334) and regulating membrane protein diffusion (78, 335, 336, 337, 338). In the absence of a vascular system, these organizational cues become important for the development of the microcirculation system, which underpins lens homeostasis and SRO and therefore preserves optical function over the lifetime of the lens.

The specialization of the membranes includes their unique lipid composition that favors transparency, symmetry, and stability (323). In lens membranes, the cholesterol:phospholipid ratios are among the highest in the body (322, 339, 340). High cholesterol levels improve stability by reducing membrane fluidity and increasing stiffness (323, 341). The stiffness of the membranes can protect lens cells against lysis, abnormal invaginations, vacuolization, and even viral infection that could disrupt symmetry, transparency, and optical function. Elevated membrane cholesterol resists oxygen diffusion to generate a protective anaerobic environment (342, 343). Membrane stability and structure is further supported by upregulation of the membrane channel/transport and adhesion proteins protective of the lens symmetry and transparency (48, 277, 296).

Historically, the realization that differentiating lens fiber cells contain an extensive cytoskeleton changed the view that lens cells were simply crystallin bags needed to generate the required “n” (73, 124, 327, 344, 345, 346, 347, 348, 349, 350). Up to 10% of the cytoplasmic protein in the lens is actin, intermediate filaments, beaded filaments, tubulin and their associated proteins, which is more varied than most other cells with the exception of muscle fibers, neurons, sperm, or actively dividing cells. The discovery of cytoplasmic microtubules in elongating fiber cells of the chick eye lens seemed to suggest their importance in fiber cell elongation (73, 351, 352, 353, 354). Instead, experimental results have found that fiber cell elongation occurred in the chick in the presence of inhibitors of microtubule assembly (355). In the mouse lens, elongation was poorly correlated with volume regulation (356) suggesting an indirect mechanism, possibly involving microtubule-mediated aquaporin or connexin functions in the regulation of hydration and osmotic stress (357, 358, 359, 360), or involvement of the cytoskeleton (76, 124) in FGF signaling (361). The alignment of the cytoskeleton to the plasma membranes and integral membrane proteins provides stability for elongating lens fiber cells (73, 124, 327, 328, 344, 345, 346, 347, 348, 349, 350, 354, 362).

In addition to this structural function, one of the lens intermediate filament proteins, the beaded filament structural protein 1 (BFSP1, aka filensin) regulates the activity of the major lens fiber cell water channel, aquaporin 0 (AQP0; (363, 364, 365)). It therefore directly regulates the optical properties of the lens (see later). BFSP1 and its intermediate filament protein coassembly partner, beaded filament structural protein 2 (BFSP2, aka CP49) together form filaments with a characteristic beaded morphology because of their association with α-crystallin. This assembly earned them the name “beaded filaments” (344, 366, 367, 368). The importance of beaded filaments to the optical properties of the lens was realized when the lens phenotype of the mouse *Bfsp2* knockout was reported. This produced a paradigm shift in our understanding, as it was the first time that lens transparency and optical function could be separated experimentally from the cataract phenotype in an animal model (369). These results led to the discovery of the function of beaded filaments in establishing emmetropia (20/20 vision), a key optical transition point for the eye (370, 371, 372), and in preserving both the optical properties and crystallin stability in the lens (32, 124, 366, 369). The protective function of BFSPs extends to scaffold formation in the establishment and stabilization of SRO and demonstrates the significance of the cytoskeleton in the development and maintenance of transparency and visual function with age ((124, 366, 369, 373, 374, 375); Fig. 7). The importance of BFSPs in visual function is an exciting and emerging new area of research (376, 377) with potential application to other protein aggregation diseases of aging and their relevant intermediate filaments (378, 379, 380). The mouse knockout of BFSP2 produces a dramatic plasma membrane phenotype. The iconic hexagonal shape of the lens fiber cell is lost and replaced with an irregular cell profile, which translates into lost transparency and optical function (369). The shape as well as the biomechanical properties of the lens is altered (381).

Intermediate filaments are indeed integral to the subplasmalemmal coat juxtaposed with the plasma membrane of lens cells (382, 383, 384). Intermediate filaments, along with the cortical actin array (385), provide mechanical and organizational support in the form of microridges (279, 280, 281, 282) to the plasma membrane (328) and its integral membrane proteins, such as AQP0, a key water channel protein (363). In the latter case, one of the lens intermediate filament proteins, BFSP1 (filensin), contains a C-terminal binding site (386) adjacent to a cryptic myristoylation site in BFSP1 (filensin) that is revealed by proteolysis. This BFSP1 C-terminal fragment was shown to regulate AQP0. BFSP1 is therefore directly involved in regulating water transport in the lens (363, 387). Indeed, the genetic removal of either beaded filaments or AQP0 phenocopy each other (365). The AQP0-beaded filament complex also affects connexin function (364), indicating that the beaded filament cytoskeleton along with water and ion channels is directly involved in the lens microcirculation system (48, 262) to regulate transparency and optical function (366, 369). Taken together, the results show the importance of the cytoskeleton in all aspects of lens cell function at all stages of development and cell differentiation (mitosis to maturation, solubility to transparent SRO, and membrane stabilization and microcirculation). We conclude that the cytoskeleton is necessary for the establishment and maintenance of noncrystalline, glass-like, transparency during development and aging in the lens (Figs. 1*B* and 7).

## Genetics and lens transparency

Many mutations are associated with cataracts in human and animal models (120, 304, 388, 389), and the list grows as more genome-wide associated studies (390) and whole exome sequencing (391) are published. From twin studies (165, 392, 393), it is apparent that both genetic and environmental factors influence ARC and longevity in general (394, 395, 396). Most cataract-causing mutations induce changes in hydration, protein conformation, and protein–protein interactions that alter microcirculation, metabolism, loss of organelles, and the establishment of SRO. Still, the genetic regulation of lens transparency and longevity in optical function remains elusive, although some key players such as DNA methylases (397) and RNA-binding proteins are clearly involved (398). The GRNs required for lens development still need to be identified (33, 399, 400, 401). The evolution of lens crystallins, their interactive sequences, and PTMs necessary for stability, refractive properties, and SRO (90, 213) need to be characterized to understand lens transparency and optical function. This review has highlighted the factors necessary for transparency and aging in the lens. Just like all other ectodermal derivatives, the lens epithelium scatters light and is not transparent (402, 403). GRNs must coordinate the proliferation, migration, and onset of elongation to assemble the growth shells of transparent lens fiber cells required for development and maintenance of optical function for the lifetime of an individual (26). The cells in the lens epithelium and particularly those in germinative and transitional zones of lens epithelium (300, 404) hold important insights to molecular and cellular aging in general, not covered by the recent discovery of a proposed common somatic mutation rate across species (405). The lens has protein preservation mechanisms in place, in addition to genetic-based aging strategies. The fundamental questions are, “What are the genetic factors that generate the only transparent cells in the mammal?” and “What factors account for remarkable molecular and cellular longevity?” Research tools exist to identify these unique GRNs to answer these questions (398, 401, 406, 407, 408).

## Summary

**Figure 8:**
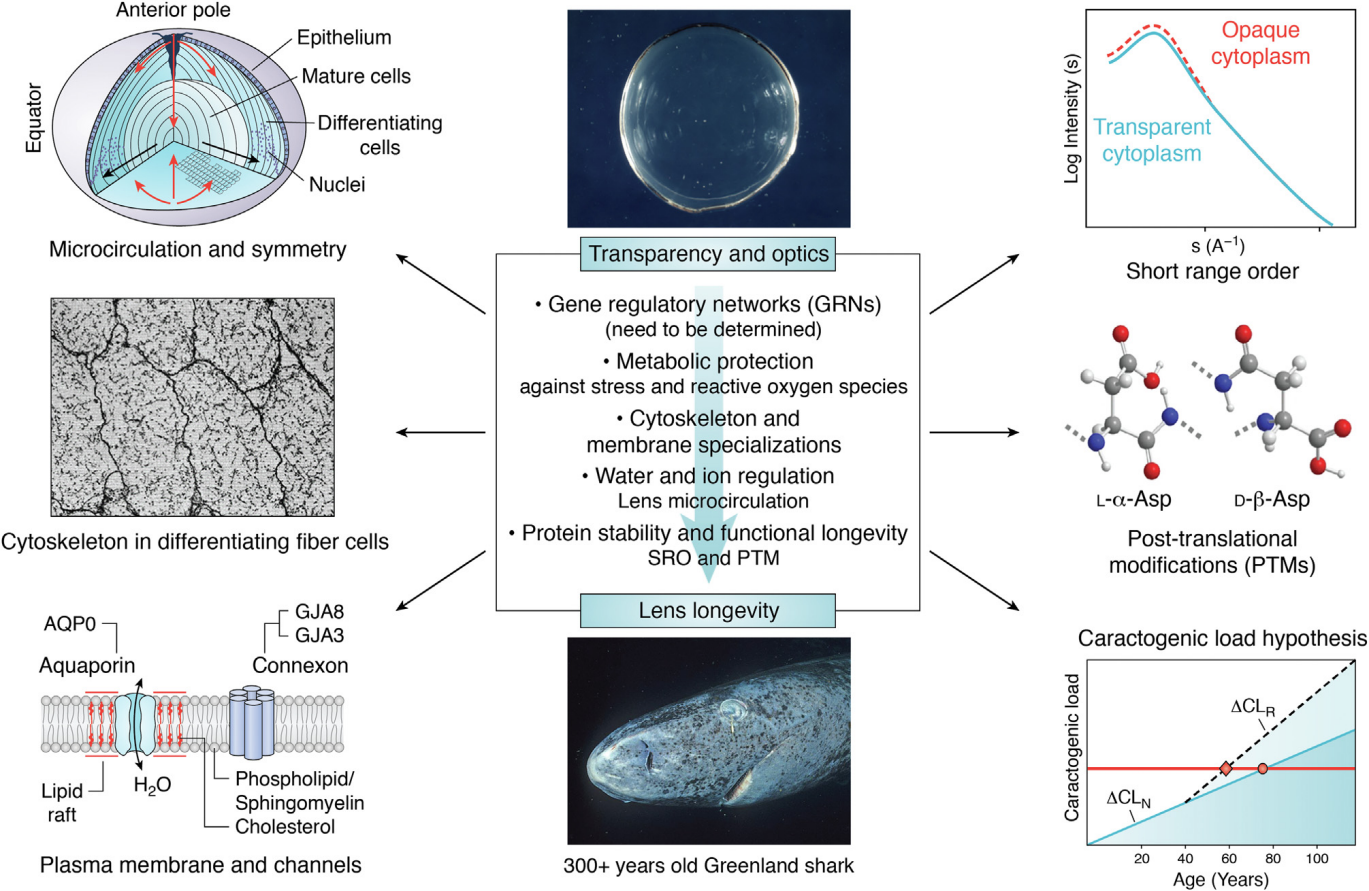
**Key factors in the development and aging of the transparent lens.** The systematic review of recent and past advances in lens and cataract research provides insight into the significance of biochemical and biophysical control of lens short-range order (SRO), transparency, and symmetry during aging. Note: While image formation is largely a function of symmetry and refractive index, transparency is linked closely to membrane, protein, and cytoplasmic structure, supported by a unique microcirculation, metabolism, and biochemistry. These factors regulate cell proliferation, migration, and elongation during differentiation and prolong the longevity of molecules and transparent cells isolated within the capsule, the thickest basement membrane in any mammalian tissue (102, 104, 124, 184, 290, 342, 423, 424). Individual panels are taken or adapted from Refs. (104, 131, 425, 426); https://commons.wikimedia.org/wiki/File:Greenland_shark_profile.jpg). DF, differentiating fiber cell; MF, differentiated and mature fiber cells.

A summary of key factors needed in the development and maintenance of transparency and optical properties in the aging lens is seen in Figure 8. Interactions at the molecular and cellular levels account for the formation of the transparent symmetric optics that contribute to the refractive properties of the living “refracton” in the eye. Their significance for visual function cannot be overstated. From the perspective of global health, these factors can impact progress on novel therapeutics to preserve and improve vision, specifically in aging populations. As a challenging scientific problem, the biology of interactions between light waves and organic matter is the basis of transparent extracellular and cellular tissues. Systematic studies are providing new knowledge about membrane, cytoskeletal, and cytoplasmic adaptations, which generate the image-forming refracton and how it is maintained and matures over a lifetime. The signaling mechanisms that synchronize proliferation, migration, elongation, and maintenance as a highly symmetric optically transparent tissue are unique to the lens. The lens is a valuable model for the study of both cellular and molecular longevity. This is particularly true with respect to protein stabilization mechanisms involving PTMs, because the very long-lived proteins are not replaced as in other nontransparent tissues.

*In utero*, lens GRNs establish the developmental, biochemical, and cell biological pathways needed to support a structure optimized for transparency and optical function for a lifetime. Whilst age expectancy can be affected by altering the environmental risk (395), it has recently been discovered that somatic mutation rate scales with lifetime (405), suggesting that this is a key factor in life expectancy. Epigenetic targets are also important (409) and make it clear that lifestyle/environment are important to a complete understanding of cellular and molecular aging (410), and that system resilience is key (411). Even for centenarians, the genetic advantages are lost later in life (412). For lens transparency, the endogenous resilience to aging and ARC includes crystallin SRO and the accumulation of PTMs to maintain it, as well as all the other biochemical, biophysical, cell biological, and metabolic adaptions of the aging lens (Fig. 8). Aging in an adult eye can be followed longitudinally by modern spectroscopic technologies (225) to correlate optical quality at the cell and molecular levels *in vivo* with mass spectroscopic imaging (141, 413, 414, 415). MRI can measure the impact of channel function or PTMs on symmetry and transparency (416). These and other technological advances can support and lead to new therapeutic approaches e.g. (417, 418) to protect image formation and improve the quality of life for the elderly. A lens is much more than an optical element in the visual system. The lens is a unique resource to characterize the environmental, metabolic, and genetic events, both positive and negative, that are experienced by proteins as a biological system ages over a lifetime.

## Conflict of interest

The authors declare that they have no conflicts of interest with the contents of this article.
